# Generation of Two Noradrenergic-Specific Dopamine-Beta-Hydroxylase-FLPo Knock-In Mice Using CRISPR/Cas9-Mediated Targeting in Embryonic Stem Cells

**DOI:** 10.1371/journal.pone.0159474

**Published:** 2016-07-21

**Authors:** Jenny J. Sun, Russell Ray

**Affiliations:** 1 Department of Neuroscience, Baylor College of Medicine, Houston, Texas, United States of America; 2 McNair Medical Institute, Baylor College of Medicine, Houston, Texas, United States of America; Ohio State University Comprehensive Cancer Center, UNITED STATES

## Abstract

CRISPR/Cas9 mediated DNA double strand cutting is emerging as a powerful approach to increase rates of homologous recombination of large targeting vectors, but the optimization of parameters, equipment and expertise required remain barriers to successful mouse generation by single-step zygote injection. Here, we sought to apply CRISPR/Cas9 methods to traditional embryonic stem (ES) cell targeting followed by blastocyst injection to overcome the common issues of difficult vector construction and low targeting efficiency. To facilitate the study of noradrenergic function, which is implicated in myriad behavioral and physiological processes, we generated two different mouse lines that express *FLPo* recombinase under control of the noradrenergic-specific Dopamine-Beta-Hydroxylase (DBH) gene. We found that by co-electroporating a circular vector expressing Cas9 and a locus-specific sgRNA, we could target FLPo to the DBH locus in ES cells with shortened 1 kb homology arms. Two different sites in the DBH gene were targeted; the translational start codon with 6–8% targeting efficiency, and the translational stop codon with 75% targeting efficiency. Using this approach, we established two mouse lines with DBH-specific expression of *FLPo* in brainstem catecholaminergic populations that are publically available on MMRRC (MMRRC_041575-UCD and MMRRC_041577-UCD). Altogether, this study supports simplified, high-efficiency Cas9/CRISPR-mediated targeting in embryonic stem cells for production of knock-in mouse lines in a wider variety of contexts than zygote injection alone.

## Introduction

Toward knock-in transgenic mouse line production, targeted double stranded breaks have emerged as a powerful tool to increase rates of homologous recombination through homology-directed repair (HDR). The CRISPR system offers simplified sequence targeting for HDR and is comprised of a Cas9 nuclease that is guided to specific target sites by a short sgRNA complementary to the target sequence followed by a PAM motif (NGG). CRISPR strategies have been used with high efficiency for knockout mutations, small deletions, and oligo mediated HDR in mouse zygotes in a potentially shorter time frame. However, successful zygotic knock-ins and germline transmission of large targeting cassettes [1–7] remains a challenge for many labs, and has yet to be widely adopted as the equipment and expertise needed for successful pronuclear or cytoplasmic injection presents a significant barrier and large numbers of mice must be screened for each targeting event.

While more time consuming, embryonic stem (ES) cell targeting and morula aggregation or blastocyst injections remains a common and at times more forgiving path to mouse line generation, particularly for low frequency targeting events or vectors of high complexity where large numbers of ES cell colonies can be rapidly screened. However, traditional ES cell targeting can also benefit from continued optimization, particularly when working with large or complex targeting vectors that are difficult to construct and work with *in vitro*, and show low rates of targeting efficiency. Toward addressing these problems, we have applied Cas9/CRISPR mediated HDR methods to ES cell targeting for large construct knock-in mouse generation. Since Cas9 mediated double stranded breaks have been shown to stimulate HDR with smaller homology targeting arms than traditional gene targeting vectors in several cell types, targeting vector construction becomes much simpler using only PCR products joined by isothermic cloning [8] and in our hands, a single ES cell electroporation has yielded multiple targets.

In our initial application of CRISPR/Cas9 integrated ES cell targeting, we chose to generate two FLPo mouse lines specific to Dopamine-Beta-Hydroxylase (DBH) expression. DBH is a key noradrenaline synthesizing enzyme and marker of brainstem and peripheral noradrenergic neurons and noradrenaline producing cells which are critically involved in a variety of behavioral and physiological processes including learning and memory, fear, anxiety, cardiovascular and respiratory function, arousal, mood, attention, and appetite, among others [9–27]. Within the brainstem, a small and diverse noradrenergic neural population projects throughout the central nervous system and is traditionally divided spatially into several anatomically distinct nuclei: the largest and most-studied A6 or locus coeruleus, and the remaining A1-5, A7, and C1-3 which also produce adrenaline. Because of the wide-spread and blurred boundaries of brainstem nuclei and the shared genetic markers between noradrenergic CNS and PNS populations, it is becoming increasingly clear that intersectional genetic approaches are needed to cleanly access and functionally study distinct noradrenergic subpopulations. While a DBH-FLPo mouse line has been reported and used in intersectional fate-mapping to show anatomical, but not functional heterogeneity [28], it is not readily available. Additionally, this mouse line is a knock-in allele that results in DBH loss of function, which may result in subtle phenotypic variations in sensitive behavioral and physiological assays.

To genetically access noradrenergic populations and demonstrate the feasibility of using CRISPR/Cas9 in ES cell gene targeting while maintaining pluripotency and germline transmission capabilities, we aimed to produce two DBH targeted FLPo mouse lines, one that is a FLPo knock-in into the DBH start codon, resulting in a null allele, and a second that is a p2a-FLPo knock-in into the 3’ end of the DBH coding sequence, leaving the DBH locus otherwise unperturbed.

## Materials and Methods

For cardiac perfusion mice were euthanized with an overdose of anesthetic (i.e. Beuthanasia D 390 mg/ml at the lethal dose of 200 mg/kg IP). The pedal reflex was checked to ensure the mouse was fully anesthetized.

Studies were approved by Baylor College of Medicine Institutional Animal Care and Use Committee under protocol AN-6171.

### Construction of Targeting and Cas9 Vectors

Targeting vectors were constructed using standard cloning procedures. For the start codon knock-in mouse line (hereon known as DBH-FLPo) targeting vector, the 5’ homology arm is a 1kb fragment going from -1000 to +0 relative to the ATG of the gene. The 3’ homology arm is a 1kb fragment extending from +106 to +1105 position (mimicking a previous knock-out strategy [29] and deleting the sgRNA sequence). For the stop codon knock-in mouse line (hereon known as DBH-p2a-FLPo) targeting vector, the 5’ homology arm is a 1kb fragment going from -1000 to 0 relative to the stop codon of the gene and the 3’ homology arm is a 1kb fragment extending +4 to +1003 (deleting the PAM motif of the sgRNA). The homology arms, FLPo or p2a-FLPo and Lox2722-flanked neomycin resistance cassettes were PCR amplified and the final targeting vector was assembled by Gibson assembly into a backbone containing a p15a origin of replication and an ampicillin selection cassette.

For the Cas9 expressing vector, we selected sgRNA target sequences that were close to the start or stop codons of the DBH gene locus. Each selected sgRNA (DBH-FLPO: CGTACATGGAAGCCGCCTCA with PAM motif CGG; DBH-p2a-FLPo: AGAATAGCTTCTCACAAGGT with PAM motif GGG), was cloned into the BbsI sites of the px330 vector [30] that expresses Cas9 (final vectors are called px330_DBH_sgRNA1 and px330_DBH_sgRNA2).

Sequences of the targeting vectors and px330 vectors have been uploaded to GenBank (KX151728, KX151729, KX151730, KX151731) and plasmids are available upon request.

### Generation of Knock-in Mice

Embryonic stem (ES) cells (AB2.2) were electroporated with 15–20 ug of varying ratios of the px330_DBH_sgRNA vector (Table 1). Neomycin selected clones were screened for homologous recombination using PCR genotyping from FLPo to the region outside the homology arm. Targeted clones were identified using PCR genotyping for 5’ and 3’ targeting from outside the homology arm to within FLPo. For the DBH-FLPo line, we used pairs 5’ CCTATCTGCTTTCCAGAGCAG (DBH-F1) and 5’CACAGGATGTCGAACTGGCTCATCACCTTC (FLPo-R1) (producing an 1164 bp band), and 5’CGGATAAAGAAACCATCTCTTCTCTT (DBH-R1) and 5’ACCTGAGCAGCTACATCAACAGGCGG (FLPo-F1) (producing a 3216 bp band). For the DBH-p2a-FLPo line, we used pairs 5’CGTGCCCTGGAACTCTTTC (DBH-F2) and FLPo-R1 (producing a 1382 bp band) and 5’CCCCATCTCTCAGGCTGTAC (DBH-R2) and FLPo-F1 (producing a 3127 bp band). To genotype for homozygosity in the DBH-p2a-FLPo targeted ES cells, we used pair DBH-F2 and DBH-R2 which produces a 2540 bp band (wt) and/or a 5670 bp band (targeted). Targeted clones were microinjected into C57B1/J6 blastocysts and chimeric males were bred to wildtype C57B1/J6 females to achieve germline transmission.

**Table 1:** Targeting efficiencies and homozygosity for FLPo knock-in into the DBH locus using CRISPR/Cas9 methods for ES cell electroporation. Shown are targeting efficiencies based on the locus and ratios of Cas9 and sgRNA expression vector: targeting vector used.

Gene locus	px330_sgRNA: targeting vector molar ratio	# of targets	Homozygosity
DBH-FLPo	0:1	0/48 (0%)	
DBH-FLPo	0.5:1	0/48 (0%)	
DBH-FLPo	1:1	4/48 (8.3%)	
DBH-FLPo	10:1	3/48 (6.25%)	
DBH-p2a-FLPo	10:1	36/48 (75%)	3/48 (6.25%)

### Breeding, Genetic Background, and Maintenance of Mice

We maintained colonies of all of our mouse strains by backcrossing to C57BL/6J mice. For routine genotyping, we carried out PCR amplification of DNA from ear punch preparations using the boiling alkaline lysis procedure [31]. FLPo-specific genotyping primers were: 5’-CACGCCCAGGTACTTGTTCT and 5’CCACAGCAAGAAGATGCTGA and yield a 226 bp band.

To achieve germline deletion of the Lox2722-flanked neomycin cassette, we crossed DBH-FLPo and DBH-p2a-FLPo males with congenic Cre-deleter females (B6N-FVB-Tg(ACTB-Cre)2Mrt). For fate-mapping of FLP expression, we crossed DBH-FLPo and DBH-p2a-FLPo heterozygotes with homozygous RC::FEE mice that conditionally express EGFP upon FLPo recombination [32]. Rosa26 specific primers for the RC::FEE mice were 5’-GCACTTGCTCTCCCAAAGTC, 5’-GGGCGTACTTGGCATATGAT, and 5’-CTTTAAGCCTGCCCAGAAGA, and yield a 495 bp band (targeted) and 330 bp band (wt).

To determine if homozygous mice were viable, we interbred heterozygous DBH-FLPo and DBH-p2a-FLPo mice and used a multiplexing locus-specific genotyping reaction for the offspring. DBH-FLPo specific genotyping primers were: 5’- GCCACTCTGCTTCGATTCTC, 5’- ACAGCAGTGCCGTACATG, and 5’- GGGATGATGGTGAACTCCCA and yield a 297 bp band (wt) and/or a 551 bp band (targeted). DBH-p2a-FLPo specific genotyping primers were: 5’-CCTAAGATCACCTCCACGCT, 5’-TCTCCGTTCAGTTGGACCTC and 5’ GGGATGATGGTGAACTCCCA and yield a 306 bp band (wt) and/or a 517 bp band (targeted).

All animal experiments were performed with the approval of IACUC. Both the DBH-FLPo (*Dbh*^*tm1(DBH-FLPo)Rray*^/Mmucd, MMRRC: 041577-UCD) and DBH-p2a-FLPo (*Dbh*^*tm2(DBH-FLPo)Rray*^/Mmucd, MMRRC: 041575-UCD) mouse lines have been accepted to the Mutant Mouse Regional Resource Center (MMRRC) and are unconditionally available to the academic research community.

### Off-Target Analysis

Off-target sequences were identified using the Optimized CRISPR Design tool (crispr.mit.edu). The top 5 sequences for each sgRNA were amplified from selected targeted ES cells and sequenced to determine if any mutations or changes occurred in off target sites.

### Immunohistochemistry and Cell Count

4–8 week old adult mice were transcardially perfused with 0.1M phosphate-buffered saline (PBS) then with 4% paraformaldehyde (PFA) in PBS. Brains were dissected out and drop fixed for two hours in 4% PFA before a PBS rinse and equilibration in 20% sucrose in PBS. Brains were then sectioned into 30 μm sections, mounted on slides, and labeled with immunofluorescent antibodies. We stained overnight with anti-tyrosine hydroxylase antibody to identify catecholaminergic neurons (1:1000, Millipore AB152) followed by 2 hours with donkey anti-rabbit Cy3 secondary (1:500, Jackson 711-165-152) in 0.1% Triton-X in PBS (PBST) with 5% donkey serum. Images were collected on a Zeiss upright epifluorescent microscope.

Co-stain cell count analysis was performed by manually counting on microscopy images the number of cells that either expressed EGFP without TH, TH without EGFP, or co-expressed EGFP and TH from 10 randomly selected images of known brainstem catecholaminergic populations from 3 mice per mouse line.

## Results and Discussion

**Fig 1:**
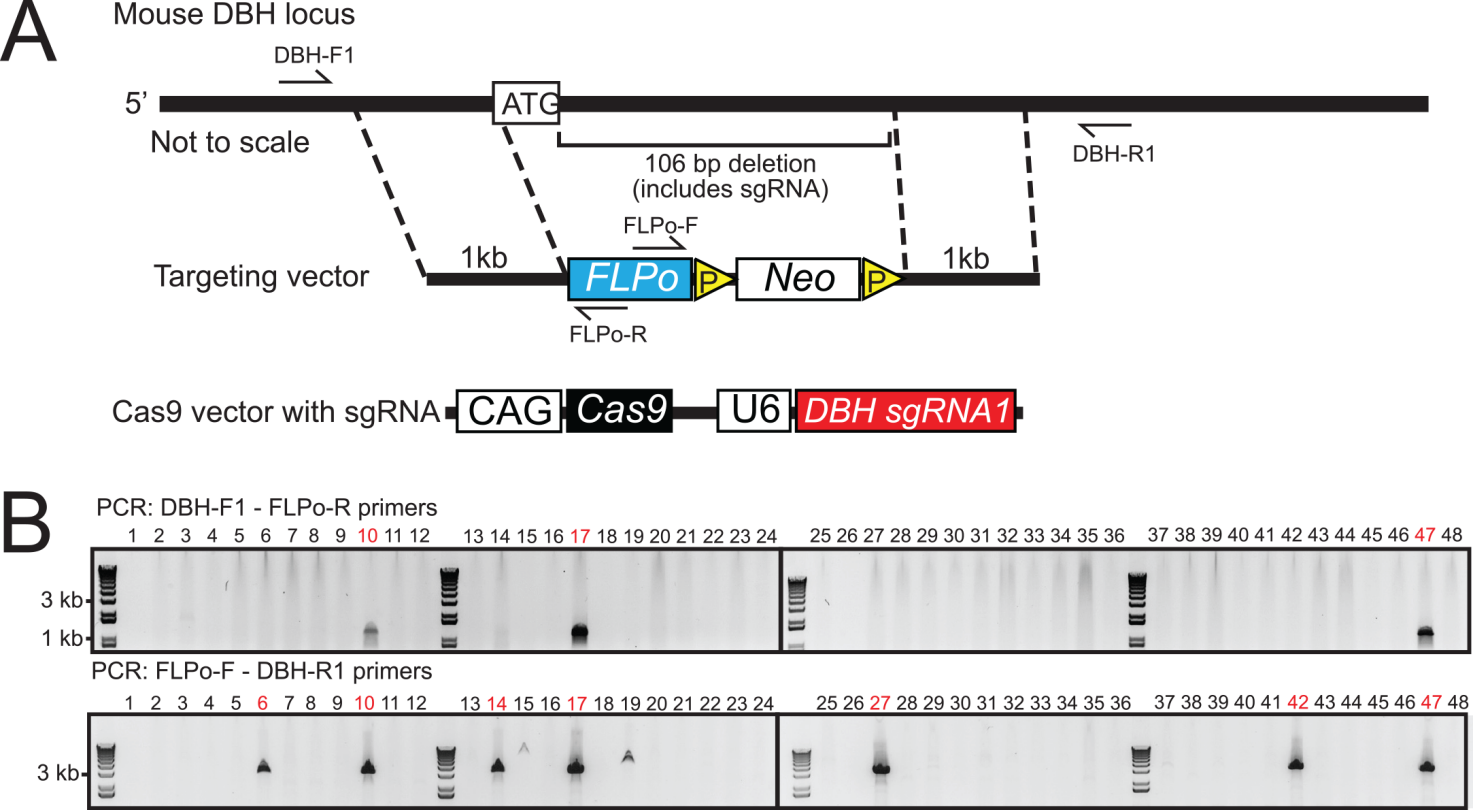
Generation of the DBH-FLPo mouse line. A) Targeting schematic. The targeting vector contains a 1kb 5’ homology arm, FLPo recombinase, Lox2722-flanked neomycin cassette, and a 1kb 3’ homology arm. The FLPo recombinase sequence and neomycin cassette was knocked into the start codon of the DBH gene, replacing 106 bps of the first exon. B) PCR genotyping of neomycin selected ES cell clones. Targeted knock-in was determined using PCR primers that spanned from within FLPo to outside each homology arm. Amplification of a band indicates targeting. Shown are the results from a 10:1 px330_DBH_sgRNA1:targeting vector molar ratio.

For the DBH-FLPo mouse line, the FLPo sequence was inserted into the transcriptional start site of the DBH gene. This resulted in the deletion of 106 bps of exon 1 and expression of FLPo (Fig 1) following the previously established targeting strategy in the first DBH^-/-^ knockout generated [29]. For positive ES cell clone selection, a Lox2722-flanked neomycin resistance cassette was integrated into the targeting vector, which included 1kb 5’ and 3’ homology arms. To use CRISPR/Cas9, we cloned an sgRNA found in the deletion area into the px330 vector, which expresses both Cas9 and the subcloned sgRNA (px330_DBH_sgRNA1).

In parallel, we attempted to create founder lines through direct oocyte injection, by 1) co-injection of the circular targeting vector without the neomycin resistance cassette and px330_DBH-sgRNA1 vector into the pro-nucleus; and 2) co-injection of the circular targeting vector without the neomycin resistance cassette and a px335_DBH_sgRNA1 vector that expresses a modified Cas9 nickase, also into the pro-nucleus (S1 Table). While this locus is amenable to Cas9 mediated gene targeting with our vectors as evidenced by our ES cell experiments described below, our attempts yielded several transgenic but no targeted founders.

Thus, we pursued the traditional ES cell electroporation route while integrating CRISPR/Cas9 to see if targeted double stranded break could increase the targeting efficiency with our reduced 1kb homology arms. We co-electroporated the px330_DBH_sgRNA1 vector with the targeting vector at four different molar ratios (0:1, 0.5:1, 1:1, and 10:1). Under the 0:1 and 0.5:1 px330 vector:donor vector conditions, we did not see any targeted clones, while under the 1:1 conditions and 10:1 conditions, we had 8.3% and 6.25% targeting, respectively (Table 1). Previously, this site in the DBH locus had been targeted with a reported efficiency of 8.3%, but with homology arms that were much longer in length [29]. This data suggests that higher ratios of the Cas9 vector resulted in increased targeting efficiency.

**Fig 2:**
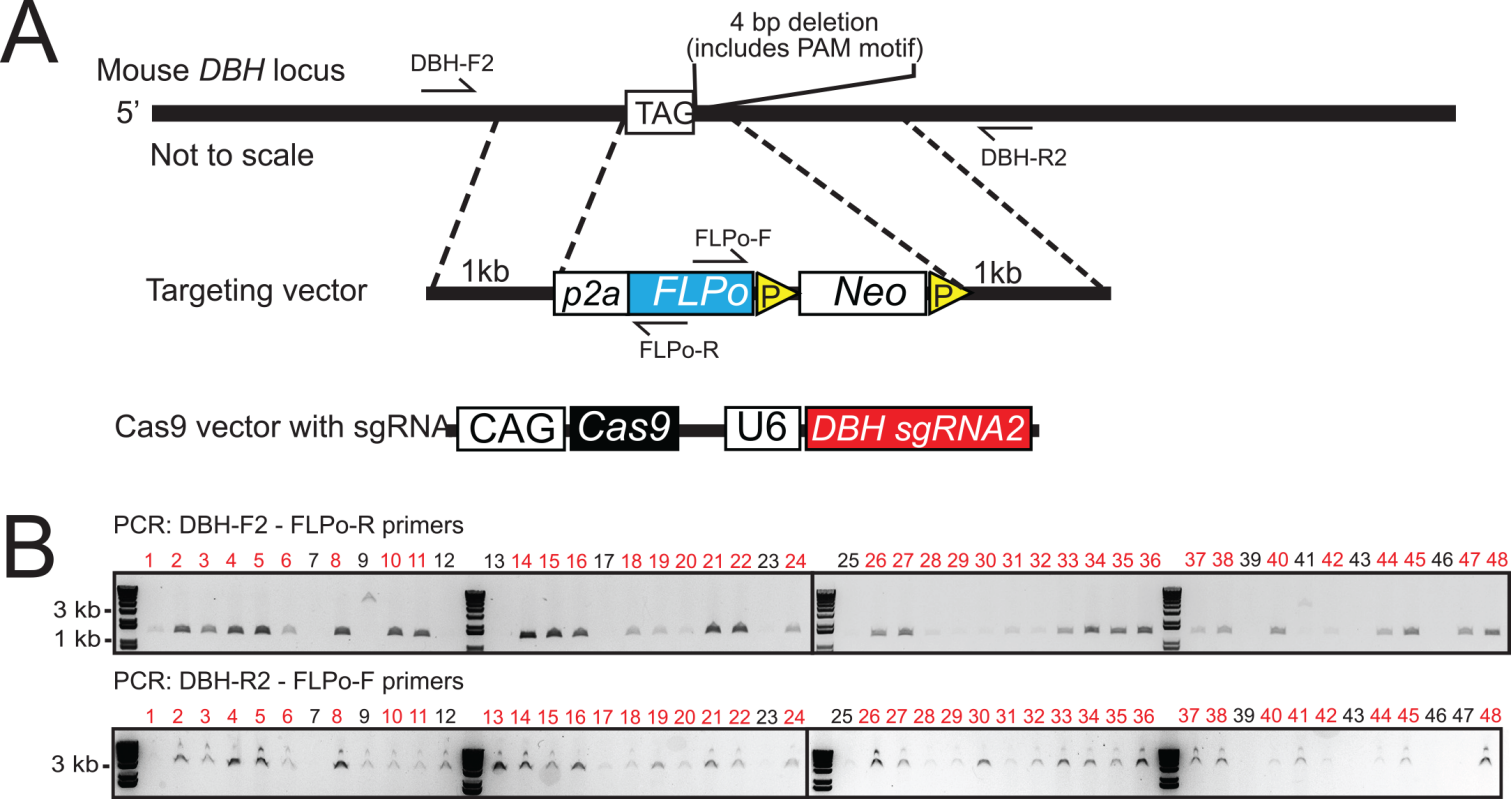
Generation of the DBH-p2a-FLPo mouse line. A) Targeting schematic. The targeting vector contains a 1kb 5’ homology arm, p2a-FLPo recombinase, Lox2722-flanked neomycin cassette, and a 1kb 3’ homology arm. The p2a-FLPo recombinase sequence and neomycin cassette was knocked into the stop codon of the DBH gene, replacing 4 bps after the last exon. B) PCR genotyping of neomycin selected ES cell clones. Targeted knock-in was determined using PCR primers that spanned from within FLPo to outside each homology arm. Amplification of a band indicates targeting. Shown are the results from a 10:1 px330_DBH_sgRNA2:targeting vector molar ratio.

While heterozygous adult DBH^+/-^ mice have no reported phenotypes, embryos during development have lower levels of noradrenaline and homozygous DBH^-/-^ mice are embryonically lethal unless rescued by administration of dihydroxyphenylserine (DOPS), a noradrenaline precursor [29]. Additionally, DBH polymorphisms in humans have been linked to altered plasma-DBH levels and a number of neurological and autonomic disorders including attention-deficit/hyperactivity disorder (ADHD) [33,34], alcohol dependence [35], post-traumatic stress disorder (PTSD) [36], migraine [37,38], and hypertension [39]. To minimize potential confounds of DBH heterozygosity and additional phenotypes that may have yet to be discovered, we also generated a mouse line where the FLPo sequence was knocked into the transcriptional stop site of the DBH gene, following a p2a self-cleaving peptide. This results in a 4 bp deletion and expression of p2a-FLPo but preserves DBH expression (Fig 2). For positive ES cell clone selection, a Lox2722-flanked neomycin resistance cassette was integrated into the targeting vector, which included 1kb 5’ and 3’ homology arms. We cloned an sgRNA with the PAM motif found in the deletion area into the px330 vector (px330_DBH-sgRNA2).

Based on our previous results suggesting that higher levels of the px330 vector resulted in higher targeting efficiencies, we co-electroporated the px330_DBH-sgRNA2 vector and targeting vector into ES cells using the highest 10:1 ratio. In strong contrast to the translational start knock-in, targeting of p2a-FLPo at the 3’ terminus of DBH gene resulted in a 12-fold increase of targeting efficiency (75%) over targeting into the 5’ terminus with the same length homology arms and cassette size (Table 1). Because the efficiency was so high, we then tested the targeted ES cells for homozygous targeting and found that 3 out of 48 colonies showed homozygosity (6.25%).

Because of the high targeting efficiency of this particular locus in ES cells, we again attempted to create founder lines through direct oocyte injection by 1) injection of *in vitro* transcribed Cas9 mRNA, transcribed sgRNA, and circular targeting vector into the oocyte cytoplasm; and 2) injection of Cas9 protein, transcribed sgRNA, and circular targeting vector into the oocyte cytoplasm (S1 Table). Although we could confirm *in vitro* that the transcribed or protein Cas9 and transcribed sgRNA complex was functional and could cut a PCR-amplified genomic fragment containing the target site, our attempts yielded several transgenic but no targeted founders.

**Fig 3:**
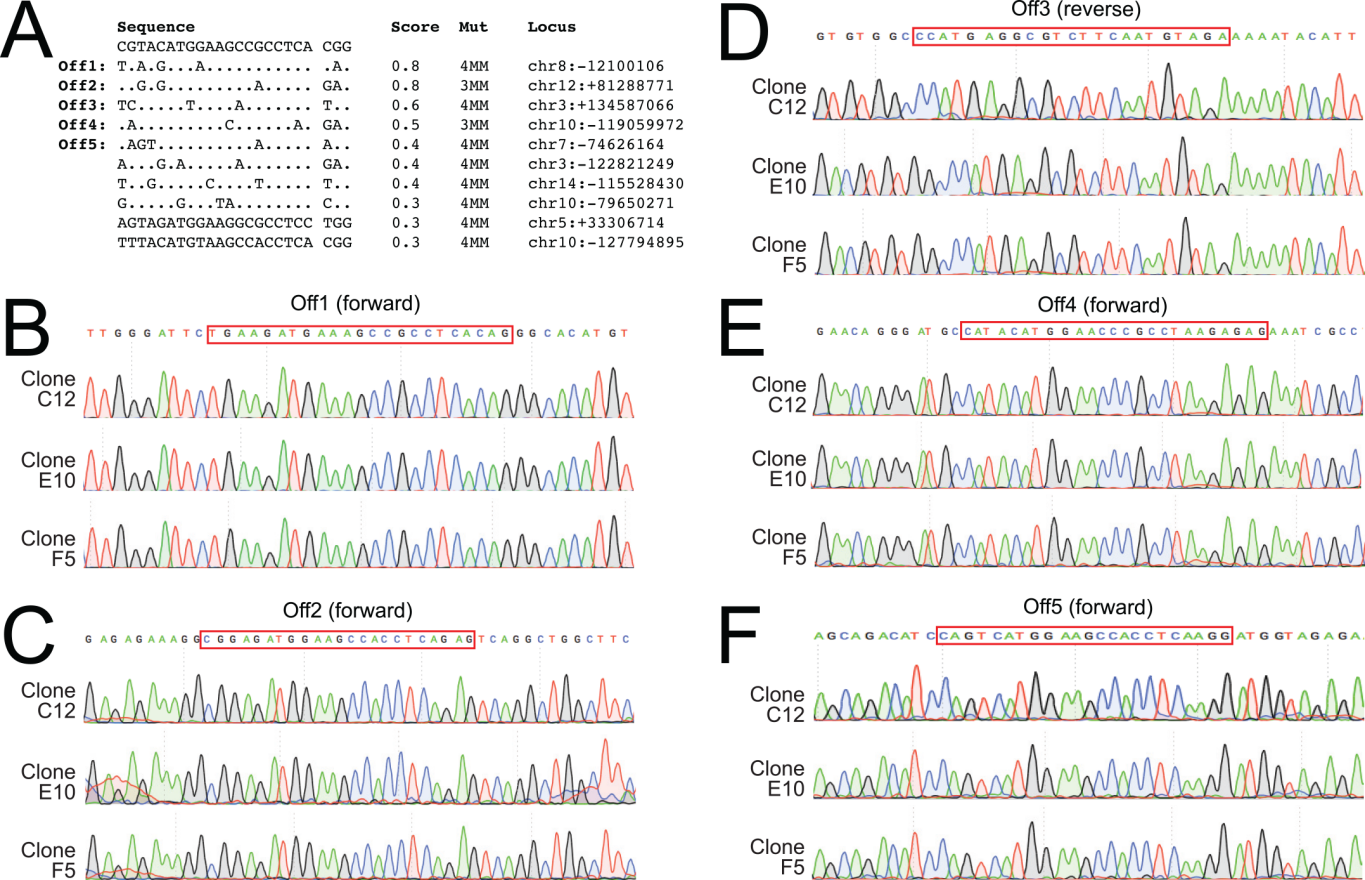
Analysis of DBH-FLPo sgRNA off-target sites. No mutations were seen in the top 5 potential off-target sites. A) List of top 10 potential off-target sites as determined by the Optimized CRISPR tool, base pair mismatches, and location in the genome. B-F) Sequence chromatograms of each off-target site, showing the correct sequence for each of the 3 selected clones that were injected into blastocysts.

**Fig 4:**
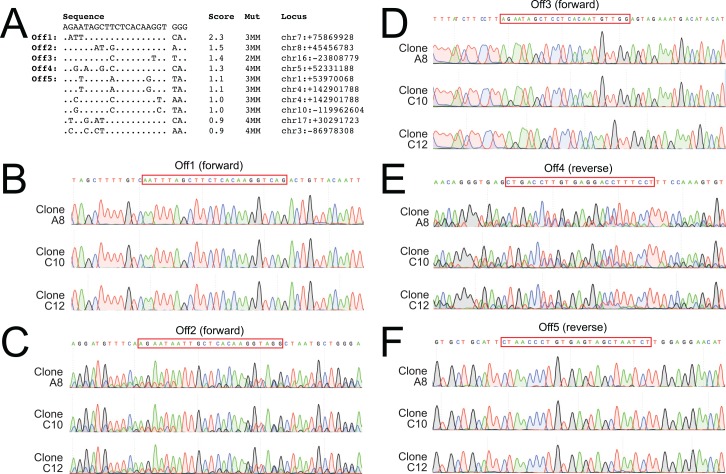
Analysis of DBH-p2a-FLPo sgRNA off-target sites. No mutations were seen in the top 5 potential off-target sites. A) List of top 10 potential off-target sites as determined by the Optimized CRISPR tool, base pair mismatches, and location in the genome. B-F) Sequence chromatograms of each off-target site, showing the correct sequence for each of the 3 selected clones that were injected into blastocysts.

Genomic sequences that are similar to the sgRNAs used for targeted double stranded break may cause unintended gene mutations or editing at off-target sites. To detect potential off-target events, we predicted the off-target sites for each sgRNA and selected the top 5 sites for follow-up. We amplified and sequenced these loci from three correctly targeted ES cell clones for each line. We did not detect any genetic modification in the DBH-FLPo sgRNA off-target sites (Fig 3) or the DBH-p2a-FLPo sgRNA sites (Fig 4). While we cannot rule out off-target effects in other loci, these data suggest that off-target effects are not dominant in this setting.

Correctly targeted clones were injected into blastocysts for chimera generation. Chimeras were mated to C57BL/6J wildtype females to confirm germline transmission. Positive offspring were mated to ubiquitous Cre deleter mice to eliminate the neomycin resistance cassette. It has been reported that heterozygous DBH^+/-^ animals have no known phenotypes. We saw no gross morphological or phenotypic deficits in our heterozygous DBH-FLPo or DBH-p2a-FLPo animals. To confirm that the DBH-FLPo is a recessive loss-of-function mutation, we interbred heterozygotes and genotyped offspring at weaning. We saw no homozygotes among 24 surviving pups (Table 2). To confirm that DBH-p2a-FLPo is not a loss-of-function mutation, we interbred heterozygotes and genotyped offspring at weaning. We found 10 out of 33 pups to be homozygous for the targeted knock-in allele.

**Table 2:** Interbreeding of heterozygous DBH-FLPo and DBH-p2a-FLPo mice suggests that DBH-FLPo is a recessive loss-of-function mutation while DBH-p2a-FLPo is not. Heterozygous DBH-FLPo mice were interbred and no homozygotes were seen among surviving weaned pups. Heterozygous DBH-p2a-FLPo mice were interbred and multiple homozygotes were seen among surviving pups.

Cross	Wildtype (25%)	Heterozygous (50%)	Homozygous (25%)
DBH-FLPo x DBH-FLPo	8/24 (33%)	16/24 (67%)	0/24 (0%)
DBH-p2a-FLPox DBH-p2a-FLPo	7/33 (21%)	16/33 (48%)	10/33 (30%)

**Fig 5:**
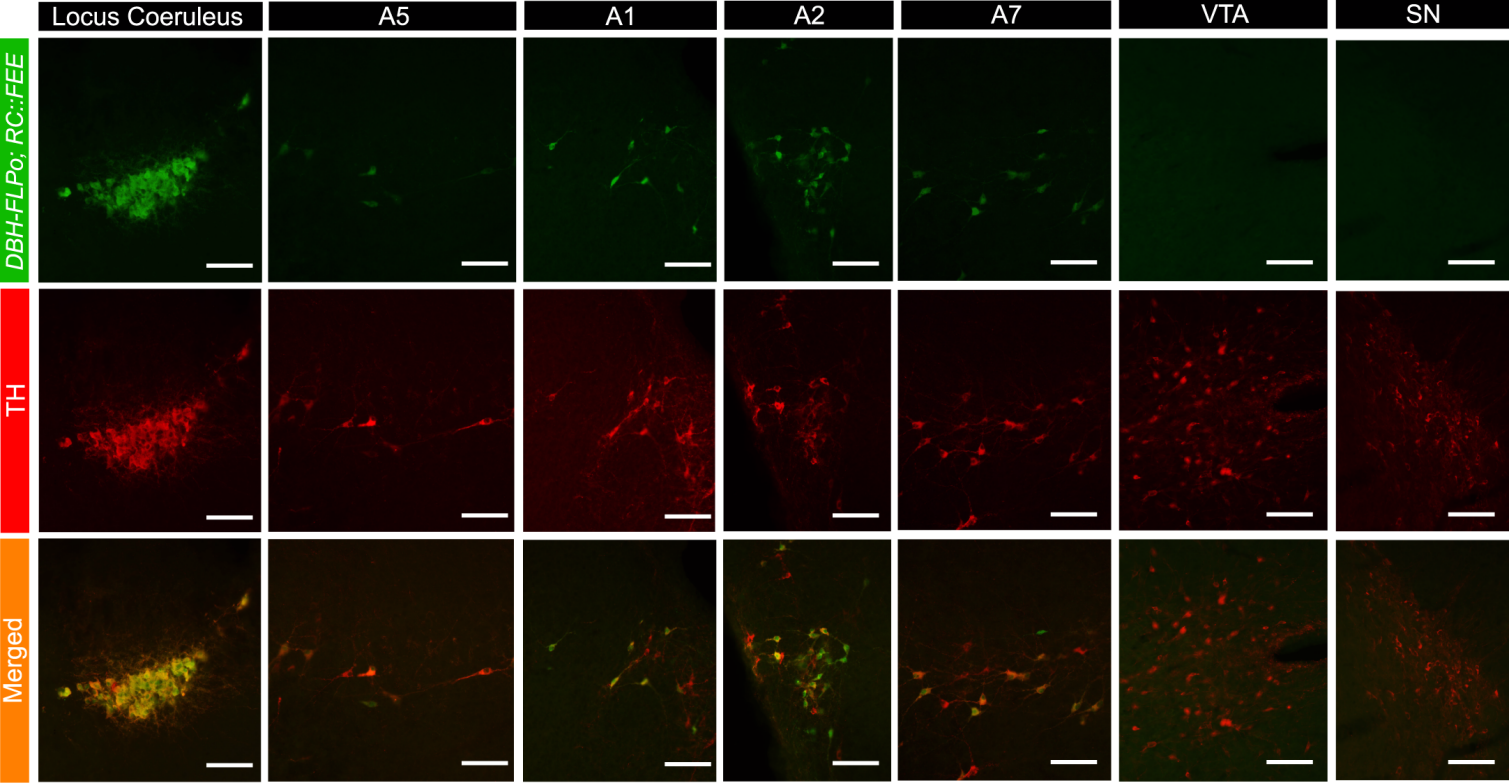
Expression of DBH-FLPo recombinase in brainstem catecholaminergic populations. Expression of EGFP in adult *DBH-FLPo; RC*::*FEE* animals in brainstem and mid-brain catecholaminergic populations. 30 μm brain sections are stained with tyrosine hydroxylase (TH, red). EGFP co-expresses with TH in all brainstem populations but is not expressed in midbrain populations. Scale bar represents 100 μm. VTA = Ventral Tegmental Area; SN = Substantia Nigra.

**Fig 6:**
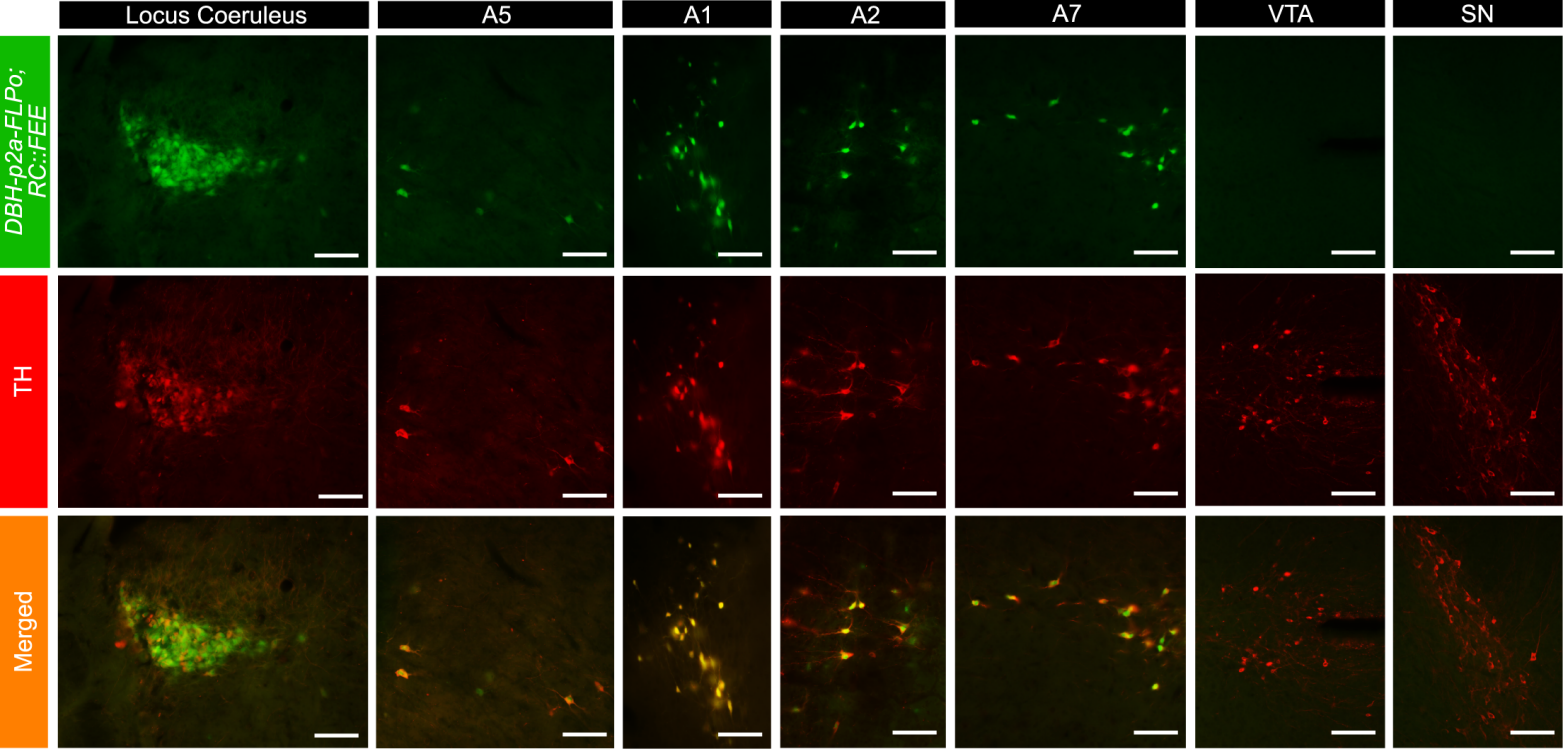
Expression of DBH-p2a-FLPo recombinase in brainstem catecholaminergic populations. Expression of EGFP in adult *DBH-p2a-FLPo; RC*::*FEE* animals in brainstem catecholaminergic populations. 30 μm brain sections are stained with tyrosine hydroxylase (TH, red). EGFP co-expresses with TH in all brainstem populations but is not expressed in midbrain populations. Scale bar represents 100 μm. VTA = Ventral Tegmental Area; SN = Substantia Nigra.

To evaluate the DBH-FLPo and DBH-p2a-FLPo recombinase activity and specificity *in vivo*, we used the R26-FRT-STOP-FRT-EGFP (RC::FEE) reporter line to reveal the expression of *FLPo* recombinase. Unlike the *TgDBH-Cre* line (which in our hands, while accurately recapitulating DBH-positive brainstem populations, also has spurious and variable expression in hippocampal and cortex neurons), expression of EGFP was limited to TH expressing populations in the brainstem, including the A6 locus coeruleus, A5, A7, and A1/C1 and A2/C2 populations (Figs 5 and 6), but not in the dopaminergic ventral tegmental area (VTA) and substantia nigra (SN) populations in the midbrain. A co-stain cell count analysis showed that most cells seen in the brainstem for both mouse lines co-expressed TH and EGFP (Table 3).

**Table 3:** Cell count analysis shows that nearly all cells in brainstem catecholaminergic populations in *DBH-FLPo; RC*::*FEE* and *DBH-p2a-FLPo; RC*::*FEE* mice co-express EGFP and tyrosine hydroxylase (TH). Cells from ten random images from 3 mice per line were manually counted for co-expression of EGFP and TH.

Mouse line	eGFP only	TH only	EGFP and TH
DBH-FLPo	2.1% ± 0.03%	2.9% ± 0.06%	94.9% ± 0.6%
DBH-p2a-FLPo	2.0% ± 0.02%	1.8% ± 0.02%	96.2% ± 0.6%

## Conclusions

In conclusion, the results reported in our study demonstrate that CRISPR/Cas9 can be used to increase targeting efficiency in embryonic stem cells with simplified, 1kb homology arms, while preserving pluripotency and the ability to transmit alleles via the germline. We have also generated two mouse lines available without restriction to the not-for-profit research community that accurately recapitulate brainstem catecholaminergic expression and can be used to investigate the role of noradrenergic neurons and cells with single and dual-recombinase responsive alleles.

## Supporting Information

S1 Table Injection conditions and outcomes for DBH-FLPo and DBH-p2a-FLPo direct oocyte injection. Plasmid DNA was purified using the GENECLEAN II kit (MP 111001400). Cas9 mRNA was transcribed using the mMESSAGE mMACHINE T7 ULTRA kit (Life Technologies AM1345) and cleaned up with the MEGAclear Transcription Clear-Up kit (Life Technologies AM1908). sgRNA was transcribed using the MEGAshortscript T7 kit (Life Technologies AM1354). Cas9 protein used was purchased from PNA Bio (PNA CB01). Injections were performed through the Baylor College of Medicine Genetically Engineered Mouse (GEM) Core.
(PDF)

S1 Fig DBH-FLPo is a loss-of-function homozygous lethal allele while DBH-p2a-FLPo preserves DBH function. A) Genotyping results from 24 intercrossed heterozygous DBH-FLPo offspring. Wt band is 297 bps and targeted band is 551 bps. No homozygous mice were seen upon weaning. B) Genotyping results from 33 intercrossed DBH-p2a-FLPo offspring. Wt band is 306 bps and targeted band is 517 bps. Ten out of the 33 pups were homozygous.
(EPS)
