# Supplementary material for: Generation of Two Noradrenergic-Specific Dopamine-Beta-Hydroxylase-FLPo Knock-In Mice Using CRISPR/Cas9-Mediated Targeting in Embryonic Stem Cells
Source: PLoS One. 2016 Jul 21;11(7):e0159474. doi: 10.1371/journal.pone.0159474 (PMC4956144; doi:10.1371/journal.pone.0159474)
Supplement: S1 Table — Plasmid DNA was purified using the GENECLEAN II kit (MP 111001400). Cas9 mRNA was transcribed using the mMESSAGE mMACHINE T7 ULTRA kit (Life Technologies AM1345) and cleaned up with the MEGAclear Transcription Clear-Up kit (Life Technologies AM1908). sgRNA was transcribed using the MEGAshortscript T7 kit (Life Technologies AM1354). Cas9 protein used was purchased from PNA Bio (PNA CB01). Injections were performed through the Baylor College of Medicine Genetically Engineered Mouse (GEM) Core. (PDF) [file pone.0159474.s001.pdf]

Gene locus	Injection location	Cas9 form	Cas9 conc. (ng/uL)	sgRNA form	sgRNA conc. (ng/uL)	Donor vector conc. (ng/uL)	# pups born	# targeted
DBH-FLPo	Pronucleus	Plasmid	5	Plasmid		10	12	0/12
DBH-FLPo	Pronucleus	Plasmid (nickase)	5	Plasmid		10	13	0/13
DBH-p2a-FLPo	Cytoplasm	mRNA	40	RNA	15	30	8	0/8
DBH-p2a-FLPo	Cytoplasm	Protein	30	RNA	20	4	23	0/23
